# Case report of severe hypoglycemic alcoholic ketoacidosis: A possible pitfall in diagnosis of ketoacidosis

**DOI:** 10.1097/MD.0000000000031996

**Published:** 2022-12-16

**Authors:** Seizo Okauchi, Fuminori Tatsumi, Kaio Takahashi, Yukino Katakura, Masashi Shimoda, Kenji Kohara, Tomohiko Kimura, Atsushi Obata, Shuhei Nakanishi, Tomoatsu Mune, Kohei Kaku, Hideaki Kaneto

**Affiliations:** a Department of Diabetes, Endocrinology and Metabolism, Kawasaki Medical School, Matsushima, Kurashiki, Japan.

**Keywords:** alcoholic ketoacidosis, forgotten medical emergency, severe hypoglycemia

## Abstract

Hypoglycemic alcoholic ketoacidosis is known to be one of the emergent diseases but its frequency is very low compared to hyperglycemic ketoacidosis or hyperosmolar hyperglycemic syndrome observed in subjects with diabetes mellitus.

**Case presentation:**

We experienced a case who had alcoholic ketoacidosis and severe hypoglycemia after drinking too much alcohol without taking enough food for a long period of time. In this subject, plasma glucose level was as low as 25 mg/dL, and ketone bodies, especially 3-hydroxybutyrate, were markedly increased. In addition, in blood gas analysis, severe acidosis was observed and anion gap was increased. These points were compatible with hypoglycemia alcoholic ketoacidosis.

**Conclusions:**

When we examine subjects with ketoacidosis and hypoglycemia, we should bear in mind the possibility of hypoglycemic alcoholic ketoacidosis especially in subjects who drink too much alcohol without taking enough food for a long period of time.

## 1. Introduction

Hypoglycemic alcoholic ketoacidosis is known to be one of the emergent diseases but its frequency is very low compared to hyperglycemic ketoacidosis or hyperosmolar hyperglycemic syndrome observed in subjects with diabetes mellitus.^[1]^ At present, there is not any clear diagnostic criteria about hypoglycemic alcoholic ketoacidosis and we have to diagnose it mainly based on medical history taking about drinking and eating. Therefore, hypoglycemic alcoholic ketoacidosis is sometimes regarded as a forgotten medical emergency.^[2]^ Also, it is well known that hypoglycemia is induced by the overuse of anti-diabetic drugs such as insulin preparation and sulfonylurea and various disorders such as insulinoma, insulin autoimmune syndrome, and adrenal insufficiency.^[3]^ In addition, hypoglycemia leads to various clinical problems such as acute coronary syndrome and fundus hemorrhage.^[4]^ It is also known that repeated hypoglycemia leads to unconscious hypoglycemia due to lack of catecholamine secretion.^[5]^ Furthermore, while the number of subjects with dementia is increasing all over the world at present which is one of the serious social issues, hypoglycemia facilitates the onset and progression of dementia especially in elderly subjects. Therefore, in clinical practice, it is very important to prevent hypoglycemia as well as to obtain good glycemic control in subjects with diabetes mellitus. Also, it is very important to precisely diagnose hypoglycemic alcoholic ketoacidosis as well as hyperglycemic diabetic ketoacidosis. Here we show a subject who had alcoholic ketoacidosis and severe hypoglycemia after drinking too much alcohol without taking enough food for a long period of time.

## 2. Case presentation

A 76-year-old Japanese female had drunk too much alcohol (200 g in ethanol equivalent) for many years without taking enough food. She sometimes felt epigastralgia and palpitation. Her appetite was decreased since last year, and thus her body weight was reduced by 8 kg these 2 years. To clarify the reason for epigastralgia and appetite loss, a gastroscopy was performed, but there were no abnormal findings. After then, since she repeatedly felt palpitation and chest discomfort, she was brought to the emergency room in our institution. In chest X-ray, there was no abnormality. In electro-cardiogram, heart rate was 106/min and frequent ventricular premature beats were observed. Her height, body weight and body mass index (BMI) were 157cm, 48.6kg and 19.6 kg/m^2^. Table 1 shows laboratory data on admission. Plasma glucose level was as low as 25 mg/dL, and hemoglobin A1c (HbA1c) was 4.8%. Insulin and C-peptide levels were very low: insulin, <0.1 μU/mL; C-peptide, 0.1 ng/mL. Ketone body levels, especially 3-hydrobuterate level, were markedly increased: total ketone bodies, 5165.4 μmol/L; acetoacetate, 1087.6 μmol/L; 3-hydroxybuterate, 4077.8 μmol/L. Various endocrine hormone levels were almost within normal range except for moderate increase of cortisol level. In blood gas analysis, severe acidosis was observed (pH 7.141) and anion gap was increased up to 23.5. Lactate level was increased up to 6.27 mmol/L. Liver and renal dysfunction was observed (alanine aminotransferase [ALT], 135 U/L; aspartate aminotransferase [AST] 45 U/L; γ-glutamyl transpeptidase [γ-GTP] 52 U/L; creatinine, 1.19 mg/dL; blood urea nitrogen, 27 mg/dL). In addition, inflammation markers were elevated: white blood cell, 17, 320/μL (neutrophil, 83.5%), although C-reactive protein (CRP) was not increased. Electrolytes were almost within normal range. Brain natriuretic peptide (BNP) was increased up to 548.1 pg/mL. Abdominal computed tomography (CT) revealed fatty liver, liver cyst, left kidney cyst but did not show any malignancy findings. Hypoglycemia is often induced in subjects using anti-diabetic drugs such as insulin and sulfonylurea, but this subject did not use any anti-diabetic agents. Since insulin level was not increased, we ruled out the possibility of insulinoma and insulin autoimmune syndrome. Also, since cortisol level was not decreased, we ruled out the possibility of adrenal insufficiency. Since we ruled out various diseases which can lead to hypoglycemia and this subject drank too much alcohol for a long period of time without enough food intake, we finally diagnosed this subject as hypoglycemic alcoholic ketoacidosis. After then, we started vitamin B supplementation as well as intravenous glucose administration to prevent possible occurrence of Wernicke encephalopathy and Korsakoff syndrome. After starting glucose administration and vitamin B supplementation, blood glucose level, ketone body level and pH were normalized within a couple of days. Also, subjective symptoms such as palpitation and chest discomfort disappeared completely, and arrhythmia was not detected at all. After recovery from hypoglycemia, there were no abnormalities in Holter electrocardiography and echocardiography. In addition, since we suspected that this subject had alcoholism, we managed her in a private room and started 20 mg of diazepam for the prevention of withdrawal symptom. We gradually decreased its dose and finally stopped it, but withdrawal symptom was not observed throughout the hospitalization period.

**Table 1:** Laboratory data on admission in this subject.

Peripheral blood		Metabolism and endocrine markers		Electrolytes	
RBC	335 × 10^4^/μL	HbA1c	4.8%	Sodium	139 mEq/L
Hemoglobin	12.1 g/dL	Plasma glucose	25 mg/dL	Potassium	4.1 mEq/L
Hematocrit	37.5%	Insulin	<0.1 µU/mL	Chloride	109 mEq/L
WBC	17230/µL	C-peptide	0.1 ng/mL	Calcium	8.6 mg/dL
Neutrophils	85.8%	Ketone bodies	5165.4 μmol/L	Phosphorus	3.1 mg/dL
Lymphocytes	12.0%	Acetoacetate	1087.6 μmol/L	Magnesium	1.9 mg/dL
Eosinophils	0.0%	Hydroxybutyrate	4077.8 μmol/L	Blood gas	
Monocyte	4.0%	TSH	0.64 µU/mL	pH	7.141
Platelet	26.8 × 10^4^/μL	FT3	2.36 pg/mL	PCO_2_	28.6 mm Hg
Blood biochemistry		FT4	0.80 ng/dL	HCO_3_^-^	9.5 mEq/L
Total protein	6.9 g/dL	ACTH	<1.5 µU/mL	Anion gap	23.5 mEq/L
Albumin	4.1 g/dL	Cortisol	40.6 µg/dL	Lactate	6.29 mmol/L
Total bilirubin	0.5 mg/dL	DHEA-S	97 µg/dL	Urine	
AST	45 U/L	Renin activity	<0.2 ng/mL/h	pH	5.0
ALT	135 U/L	Aldosterone	58.7 pg/mL	Glucose	(±)
LDH	225 U/L	Adrenaline	23 pg/mL	Protein	(±)
ALP	269 U/L	Noradrenaline	190 pg/mL	Occult blood	(−)
γ-GTP	52 U/L	Dopamine	10 pg/mL	Ketone body	(2+)
Creatinine	1.19 mg/dL	BNP	548.1 pg/mL	Specific gravity	1.013
BUN	27 mg/dL	Vitamin		Feces	
Uric acid	11.8 mg/dL	Vitamin B1	29 ng/mL	Occult blood	<30 ng/mL
Amylase	101 U/L	Vitamin B12	376 pg/mL		
CRP	0.14 mg/dL	Folic acid	7.1 ng/mL		

γ-GTP = γ-glutamyl transpeptidase, ACTH = adrenocorticotropic hormone, ALP = alkaline phosphatise, ALT = alanine aminotransferase, AST = aspartate aminotransferase, BNP = brain natriuretic peptide, BUN = blood urea nitrogen, CRP = C-reactive protein, DHEA-S = dehydroepiandrosterone sulphate, FT3 = free triiodothyronine, FT4 = free thyroxine, HbA1c = hemoglobin A1c, LDH = lactate dehydrogenase, RBC = red blood cell, TSH = thyroid stimulating hormone, WBC = white blood cell.

## 3. Discussion

We experienced a case who had alcoholic ketoacidosis and severe hypoglycemia after drinking too much alcohol without taking enough food for a long period of time. Hypoglycemic alcoholic ketoacidosis is one of the emergent diseases and can lead to sudden death,^[6]^ although there is not any clear diagnostic criteria about it and it is sometimes regarded as a forgotten medical emergency. Alteration of mental conditions and loss of consciousness are sometimes observed due to severe hypoglycemia in patients with alcoholic ketoacidosis.^[1]^

In general, in alcoholic ketoacidosis, nicotinamide adenine dinucleotide + hydrogen (NADH)/nicotinamide adenine dinucleotide (NAD) ratio is increased through ethanol metabolism, which suppresses gluconeogenesis in the liver and impairs fatty acid oxidation.^[7]^ The oxidization of ethanol to acetaldehyde is involved in the marked reduction of NAD to NADH. For re-oxidization of NADH, pyruvate is converted to lactate.^[8]^ Such process finally leads to metabolic acidosis accompanied by increased anion gap and ketone bodies, especially 3-hydroxybutyrate. It has been reported that 3-hydroxybutyrate/acetoacetate ratio is significantly higher in patients with alcoholic ketoacidosis compared to those with diabetic ketoacidosis.^[1,9]^ In general, severe acidosis is not common in patients with alcoholic ketoacidosis compared to diabetic ketoacidosis. After treatment of alcoholic ketoacidosis, hepatic NADH/NAD ratio and gluconeogenesis are normalized, which leads to decrease of lactic acid and increase of acetoacetate.^[1]^

Poor food intake decreases glycogen storage in the liver, which leads to the occurrence of hypoglycemia in patients with alcoholic ketoacidosis. In alcoholic ketoacidosis, lactate level could be increased. It has been reported that lactate levels are higher in patients with alcoholic ketoacidosis compared to those with diabetic ketoacidosis.^[1]^ In this subject, the anion gap was increased and ketone bodies, especially 3-hydroxybutyrate, were increased. Also, severe hypoglycemia (plasma glucose level, 25 mg/dL) was observed. In addition, lactate level was increased up to 6.27 mmol/L in this subject. These points were also compatible with hypoglycemia alcoholic ketoacidosis.

Taken together, when we examine subjects with ketoacidosis and hypoglycemia, we should bear in mind the possibility of hypoglycemic alcoholic ketoacidosis especially in subjects who drink too much alcohol without taking enough food for a long period of time.

## Author contributions

SO, FT, KT, and HK researched data and/or wrote the manuscript. YK, MS, KK, TK, AO, SN, TM, and KK contributed to discussion.

**Investigation**: Seizo Okauchi, Fuminori Tatsumi, Kaio Takahashi, Hideaki Kaneto.

**Validation**: Yukino Katakura, Masashi Shimoda, Kenji Kohara, Tomohiko Kimura, Atsushi Obata, Shuhei Nakanishi, Tomoatsu Mune, Kohei Kaku.

**Writing – review & editing**: Hideaki Kaneto.
